# The Microscope

**Published:** 1886-10

**Authors:** 


### *THE MICROSCOPE.*

No two instruments have played a more important part in science than the telescope and the microscope. They have done more to revolutionize old theories founded in error, and build anew upon foundations of ascertained truth, than nearly all others put together. It is comparatively of recent date that the dominant ecclesiastical powers enjoined, nay, *enforced* as an essential article of belief, the assumption that the globe we inhabit was the center of the material universe, transfixed in the heavens, around which all other worlds revolved, and to which they were in a manner tributary; but the telescope has revealed millions of worlds, with their sidereal groups, systems and suns, and made the names of Galileo, Herschel and Kepler famous for all time; while the microscope has been equally efficient in revealing those minute forms of swarming life all about us, which the unaided eye is powerless to discern. Though in point of time the junior of its big brother, the microscope occupies a field of usefulness scarcely less in importance to the telescope, for it is at once pioneer, instructor and a safeguard in nearly all sanitary measures and remedial agents. Its astonishing disclosures will be appreciated when it is considered that the mites in cheeses were formerly thought to be the minutest forms of animal existence, while it is now computed that a single mite is twenty-seven million times larger than some of the animaculæ brought to light by the microscope, some of those discernible in certain water being so small that a thousand millions of individuals, each with a distinct organism, occupy no more space than a grain of sand.

While the improvements in microscopes have kept pace with those of other optical inventions, any new discovery which will tend to its still higher development, cannot fail to be of interest to our readers, and it is with no small degree of satisfaction that we append the following account of a recent discovery in the manufacture of glass adapted to microscopic lenses, which will greatly increase their refractive qualities, extracted from one of our foreign exchanges: "It was in the year 1878 that Dr. Abbey read a paper before the South Kensington Association, in England, in which he strongly set forth his ideas that it was possible that a new kind of glass might be made which would increase the power of the microscope. In the year 1881 he and Dr. Scott, a celebrated chemist, began to experiment in the town of Witten, in Westphalia. Their funds gave out in 1883, however, before they had obtained any remarkable results from their experiments, but both were so sanguine of making a beneficial discovery if they had funds to continue their work that the Prussian Government was induced to give $1,500 for the continuation of experiments. Professor Abbey and Dr. Scott then erected a laboratory in Germany, at the works of Carl Zeiss, a manufacturer of instruments used by scientists. They tried nearly all known elements, and it was only about six months ago that their long labor was crowned with success, which will make them famous in the history of science.

"The ordinary glass contains six substances. The new glass made by Prof. Abbey and Dr. Scott contains 14. The most essential elements of which it is composed are phosphorus and boron, neither of which is used in common glass. With the old glass the full power of the microscope was the discernment of the one-five-hundred-thousandth part of an inch, and with the new glass it is claimed that the one-two-hundred-and-four-million-seven-hundred-thousandth part of an inch can be distinguished. This certainly seems incredible, but positive assurance of its truth is given by parties who have tested Prof. Abbey's and Dr. Scott's new instrument. The fact of the experiments having been conducted with funds supplied by the Prussian Government prevent the discoverers from making it a private enterprise, and compels them to make it a public benefit. The difference between the new and the old consists in the refraction of light. The glass is not on the market yet, but will be very shortly. It will be used entirely for high-power instruments. The benefits to be derived from the discovery can hardly be estimated. It will, of course, be of great value in microscopic photography."

---

**Youth's Companion** is one of the oldest and best weekly story papers published for children, and even older people find themselves interested in its contents. Published at Boston, Mass. Price $1.75 per annum.

